# The Mayo Clinic experience: Once daily dosing of isotretinoin for acne

**DOI:** 10.1016/j.jdin.2024.07.008

**Published:** 2024-08-02

**Authors:** Hasina Maredia, Megha Tollefson, Aaron Keehr, Julio C. Sartori-Valinotti

**Affiliations:** a Department of Dermatology, Mayo Clinic, Rochester, Minnesota; b Division of Clinical Informatics and Practice Support, Mayo Clinic, Rochester, Minnesota

**Keywords:** acne, general dermatology, isotretinoin, medical dermatology, quality improvement

*To the Editor:* Oral isotretinoin was approved by the US Food and Drug Administration in 1982 for the treatment of severe, resistant nodular acne. Prescribing practices and laboratory monitoring have evolved over time. There remains a gap in consensus for isotretinoin dosing frequency. Isotretinoin (Accutane, JG Pharma Inc) medication guides recommend 0.5-1.0 mg/kg/day in two divided doses with food, stating: “The safety of once daily dosing with Accutane has not been established. Once daily dosing is not recommended.”^1^ However, increased experience with isotretinoin has now supported once daily dosing. This article’s objective is to review prescribing practices of isotretinoin for acne at Mayo Clinic Department of Dermatology, perform a subset analysis evaluating a quality improvement intervention, and review literature support for once daily dosing of isotretinoin.

The Slicer Dicer feature of Epic Hyperspace was used to examine isotretinoin prescriptions between January 2018 and March 27, 2024. Descriptive data included total number of prescriptions and distinct patients, patient demographics, and frequency of once daily versus twice daily dosing (equivalent total daily dose divided into two doses). In May 2023, default isotretinoin orders were changed from twice daily dosing to once daily dosing. Dosing frequency was compared before and after the intervention with a chi-squared test.

A total of 6282 prescriptions of isotretinoin were written for 1714 patients over 6 years (Table I). During the entire study period, 92% (*n* = 5767) of prescriptions were once daily dosing, 7% (*n* = 453) were twice daily dosing, and 1% (*n* = 62) were indeterminate. In a subset analysis comparing the 10 months before and after the intervention, frequency of twice daily dosing significantly decreased from 9.9% (*n* = 122/1237) to 5.7% (*n* = 74/1307) (*P* < .01).

**Table I:** Clinical characteristics of patients prescribed isotretinoin at Mayo Clinic Department of Dermatology

Clinical characteristics	Values
Number of patients	1714
Number of prescriptions	6282
Mean age of patients	23 y (SD 7)
Female	49.1%
Mean once daily dose	53 mg (SD 19)
Mean twice daily dose	33 mg/dose (SD 8)

At Mayo Clinic, isotretinoin for acne is primarily prescribed once daily with the largest fat-containing meal of the day. Generic isotretinoin is lipophilic with low bioavailability and is advised to be taken with lipid-rich meals.^2^ In the fed state, mean elimination half-life is 21 hours in adults and 15 hours in pediatric patients (12-15 years).^1^ However, approximately 50% of teenage boys and 66% of teenage girls do not eat breakfast,^3^ leading to one of two doses with twice daily dosing having limited absorption.^3^ In contrast, once daily dosing in a fed state has higher isotretinoin blood levels than isotretinoin given fasting at 12-hour dosing.^2^ Daily dosing also has the advantage of improving adherence,^4^ while side effects are dependent on cumulative daily dose rather than on dosing frequency.^5^

In group practices with shared patients, a consensus on dosing frequency can help avoid prescription errors. Due to default orders originally listing twice daily dosing, at times prescriptions unintentionally were written for twice daily dosing, which significantly decreased once the default was changed to daily dosing. The isotretinoin medication guide labeling of twice daily dosing may limit institutional change of default orders in the absence of formal literature supporting once daily dosing. This article provides the literature evidence and the prescribing experience for isotretinoin at Mayo Clinic to support daily dosing. Limitations to the study include the retrospective study design, and prospective studies are warranted for assessment of safety and efficacy of dosing frequencies.

## Conflicts of interest

None disclosed.

## References

[1] (2022). MEDICATION GUIDE ACCUTANE®. http://www.rxaccutane.com/assets/pdf/accutane-pi-and-medication-guide.pdf.

[2] Webster G.F., Leyden J.J., Gross J.A. (2013). Comparative pharmacokinetic profiles of a novel isotretinoin formulation (isotretinoin-Lidose) and the innovator isotretinoin formulation: a randomized, 4-treatment, crossover study. J Am Acad Dermatol.

[3] Rampersaud G.C., Pereira M.A., Girard B.L., Adams J., Metzl J.D. (2005). Breakfast habits, nutritional status, body weight, and academic performance in children and adolescents. J Am Diet Assoc.

[4] Eisen S.A., Miller D.K., Woodward R.S., Spitznagel E., Przybeck T.R. (1990). The effect of prescribed daily dose frequency on patient medication compliance. Arch Intern Med.

[5] Daly A.U., Baptista Gonçalves R., Lau E. (2023). A systematic review of isotretinoin dosing in acne vulgaris. JEADV ClinPract.

